# Learning from traumatic experiences with brief eclectic psychotherapy for PTSD

**DOI:** 10.3402/ejpt.v4i0.21369

**Published:** 2013-12-20

**Authors:** Berthold P. R. Gersons, Ulrich Schnyder

**Affiliations:** 1 Department of Psychiatry, University of Amsterdam and Arq Psychotrauma Expert Group, Amsterdam, The Netherlands; 2 Department of Psychiatry and Psychotherapy, University Hospital Zurich, Zurich, Switzerland

**Keywords:** Posttraumatic stress disorder, treatment, learning

## Abstract

Brief eclectic psychotherapy for PTSD (BEPP) is an evidence-based therapeutic approach that combines and integrates elements from psychodynamic, cognitive-behavioral, and directive psychotherapy. Psychoeducation is done jointly with the patient and his or her partner. Exposure, a structured writing task, and memorabilia are used to help patients accessing, feeling and expressing their suppressed emotions related to the traumatic experience. In the domain of meaning stage, patients will learn how they and their view of the world have changed, and that they have become “sadder but wiser”. Much emphasis is put on the vulnerability of human beings. Finally, an individually tailored farewell ritual is done to end treatment, to reunite with loved ones, and to go on with life.

The first author designed brief eclectic psychotherapy for PTSD (BEPP) during the 1980s. Confronted with a large group of police officers in Amsterdam who had developed PTSD after shooting incidents (Gersons, 1989), he felt the need to develop some kind of effective treatment for PTSD. Well-known treatments nowadays, such as trauma-focused cognitive-behavioral therapy (CBT) (Foa, Keane, & Friedman, 2000 *)* or Eye Movement and reprocessing Therapy (EMDR) (Shapiro, 1995), were nonexistent at the time. The symptoms of PTSD such as intrusive memories, heightened arousal, and increased irritability were very prevalent in these police officers. However, the focus in the beginning of the development of the BEPP protocol was on working through the traumatic event by understanding the fear and the horror. Furthermore, much attention was given to the patients’ personal biographical background: experiences from childhood, the development of a personality, as well as their reasons to choose a career as a police officer.^1^

The same framework was used by Jack Lindy for treating traumatized Vietnam Veterans, based on a general psychodynamic approach to understand the personal tragedy of a police officer or a soldier (Lindy, 1988). The result of these treatments was positive with respect to patients’ satisfaction. Their personal tragedy was framed within what had happened to them in relation to their own unique development from childhood on. Also the avoidance symptoms decreased slightly. However, the other symptoms of PTSD like re-experiencing and hyperarousal symptoms remained unchanged. Thus, the main benefit of the psychodynamic treatment approach was that people learned about themselves, about their emotions, about the reality of danger, and the dark side of human tragedy as about their personal biographical narrative, which had laid the ground for how they coped with evil.

## Searching for effective treatment ingredients

When psychodynamic treatment did not work it was the rule at the time to find out if behavior therapy could offer a solution. In the treatment of phobias, for instance, psychodynamic treatment had shown little effect on symptom levels. However, repeated gradual exposure to the phobic situation was effective in diminishing anxiety. This finding implicated a paradigmatic change in psychotherapy. Psychodynamic treatment was based on the principle that one has to go back to one's childhood to understand the symptoms. By this process of understanding and experiencing related emotions, psychotherapy was expected to yield beneficial effects. Behavior therapy now showed positive results without paying attention to childhood issues. Patients learned to change their behavior by understanding the irrationality of fears and symptoms, and by training, with the help of the therapist, to face and ultimately overcome their fear. An important secondary gain was the frustration of being too fearful in front of certain situations, f.i. spiders. This frustration was exchanged for a renewed feeling of being in control and having confidence in oneself. These two effects together became well known as CBT. Edna Foa (Foa & Rothbaum, 1998) was among the first to adopt CBT for PTSD, especially for PTSD following rape. Where raped women with PTSD avoided having intimate relations with men, they felt more secure in starting new relationships with men after CBT.

In the treatment of PTSD, it is important to decrease the symptoms of PTSD but also to help patients to overcome the standstill in their life, being afraid to move forward, not knowing who they can trust.

Now, in the development of BEPP, a different route was taken. A cornerstone in psychodynamic treatment was the coming to the surface of hidden or suppressed emotions. The empathic attitude of the therapist or the explicit focusing on emotions like in mentalizing or mindfulness nowadays should help to surface or express the emotions. After such expressions, people would feel a sense of relief and relaxation. In the first phase of development of BEPP, such an approach was practiced with limited results. Erika Fromm (Fromm & Shor, 1979), the nestor of hypnotherapy, had well demonstrated the need for a form of concentrated exposure to events in the past to help the emotions coming to the surface. In the treatment of blocked grief reactions, confrontation with photos of the deceased was also a powerful tool in precipitating a cathartic outcry of blocked emotions (Ramsay, 1977).

These facts have created a new understanding. Most patients suffering from PTSD do not like, and actively avoid, talking about their traumatic experiences. The events are perceived as too painful. PTSD patients, and also those who hear about the events, are afraid of the emotions of grief and fury, and the fear that is stimulated when imagining the terrible situations. Very focused and concentrated confrontation with the memories of the traumatic event might help the patient accessing, feeling, and expressing their suppressed emotions. Thus, in BEPP, a combination of relaxation exercises, immediately followed by *imaginary exposure*, was introduced. With eyes closed and with the help of the therapist who stimulates the now controlled imaginary re-experience of the traumatic event, emotions such as anxiety, grief, and sorrow will be experienced very strongly. In contrast to the practice of exposure in CBT, in BEPP it is not a repeated exposure in which the same moment or event is “visited’’ again and again until all anxiety has faded. In BEPP the whole sequence of events is imagined part-by-part until all emotions related to the traumatic event have been fully experienced. Secondary to the expression of emotions in BEPP is a decrease in fear and the dissolving of PTSD symptoms.

A number of additional tools were introduced in BEPP to help express strong emotions. Just like a photo of a deceased loved one, certain objects connected to the trauma can help stimulating the emotions. For instance, clothes the patient had worn during the rape, or newspaper articles still hidden in a cupboard, can be very helpful in stimulating emotions now felt about the tragic past. In BEPP, patients are encouraged to bring those *memorabilia* to the treatment sessions. Another powerful tool is the instruction to write an angry *letter* to someone or an organization rationally or irrationally blamed for the traumatic incident. It is not a letter to actually be sent in reality but to help experiencing and accepting feelings of rage and anger related to the trauma. The letter is written in the days between the sessions and is read aloud and discussed during sessions. The writing of such letters often helps to feel satisfied expressing difficult feelings and being able to do so. Similar to CBT, the letter helps to regain some self-respect. People are often very angry to have been powerless victims of others. The expression of anger helps them in overcoming their shameful passiveness.

Before applying exposure, memorabilia and the letter writing to help the patient in accessing, feeling, and expressing their suppressed emotions, it is important to understand that people with PTSD are afraid of treatment. They also want to find out if they can trust the therapist. Is the therapist really interested in the patient, can the therapist stand all the horrific details, can he or she really understand and imagine what the patient went through? This testing phase is a key to the successful application of exposure treatment. The patient also wants to find out if the therapist is really an expert in the field of psychotraumatology.

In BEPP, this has led to a very precise form of *psychoeducation*. This is the instruction for the first session of BEPP. A partner or trusted person of the patient is also invited to come to the session and share the listening to the explanation of PTSD symptoms. These are explained in relation to the traumatic experience of the patient. Once the patient has learned what PTSD is in his or her personal experience and story, the treatment is explained in detail. Because they are so familiar with PTSD, therapists often forget that the patient has no knowledge about trauma and PTSD and does not connect symptoms to the traumatic events.

Summarizing BEPP so far, it starts with psychoeducation followed by sessions with relaxation and imaginary exposure and the use of memorabilia and letter writing. Patients and therapists often describe this phase of BEPP as a very natural process leading to the need to understand more about how the traumatic incident has changed them and their view of the world. This *domain of meaning* is a unique element in BEPP compared to other trauma-focused treatments, such as CBT and EMDR. It is about the realization that life will never be the same again as before the traumatic event. One is confronted with the question of how much one can trust nature, technology, or, even more difficult, other people. And by asking these questions one has to find out what shaped one's attitudes and expectations about oneself in childhood and later in life, about norms, values, religion, prejudice, and naivety. This is not an easy part of the treatment: much knowledge and skills on the side of the therapists are needed. The *domain of meaning* is less structured as the other parts of BEPP and it can take between 6 and 10 sessions, making BEPP longer than other trauma-focused approaches such as EMDR.

The final ingredient which has been added to BEPP is the application of a *farewell ritual*. Here we drew on the important work of Onno van der Hart (1985). Van der Hart wrote his dissertation about farewell rituals as they are used, for instance, in funerals where the body of the deceased is not present. This is the case, for example, when a fisherman has drowned at sea during bad weather. Possessions of the fisherman are used instead of the body to say farewell to the deceased. The memorabilia are powerful in helping the bereaved family members and friends realizing their loss, and stimulating emotions. In BEPP, the farewell ritual is used in the same manner. A patient is asked to conceive a plan for a farewell ritual to leave the traumatic event behind, not to forget but to be able to go forward in life. A man who survived a bombing attack on the London subway burned the photos in which he was photographed leaving the tube station with other injured survivors. Burning memorabilia also means accepting some aggressiveness toward elements related to the trauma. The patient is advised to share the farewell ritual with a partner. After the burning or some other form of symbolic destruction, cleansing, or “letting go,” there should also be an element of celebrating the ending of treatment. We know from the life events literature that rituals are important tools to overcome the emotions and to go on with life. Here, the farewell ritual is also a transitional ritual to reunite with life and loved ones.

For the therapist, a farewell ritual has an additional purpose. From experience, we know that the patient often has a high appreciation for the therapist who is able to listen and offer solace and support. This gives a strong positive transference, which is not easily stopped by the therapist. Taking this into consideration, the farewell ritual is a helpful tool for the therapist to free him or herself from the tendency to carry on with treatment. Especially in BEPP, the domain of meaning phase gives a lot of freedom to come up with many important topics to be discussed with the patient. The limitation of BEPP to 16 sessions and the farewell ritual help to limit unnecessary extensions of treatment.

## Structure of the BEPP protocol

The BEPP protocol consists of 16 meticulously predefined sessions.^1^ The therapy starts with psychoeducation; a partner or loved one is also invited to attend the first session. Psychoeducation gives the patient control and motivates them to engage in therapy. One of the characteristics of PTSD is that patients are on the alert, constantly anticipating danger. As a result, while being highly alert to potential danger, they often have great difficulties focusing on ordinary things. They are anything but forgetful where the trauma is concerned, but often forget many things that are not related to the trauma.

The 16 sessions in the protocol are structured as follows (Gersons & Olff, 2005):

**Figure:**
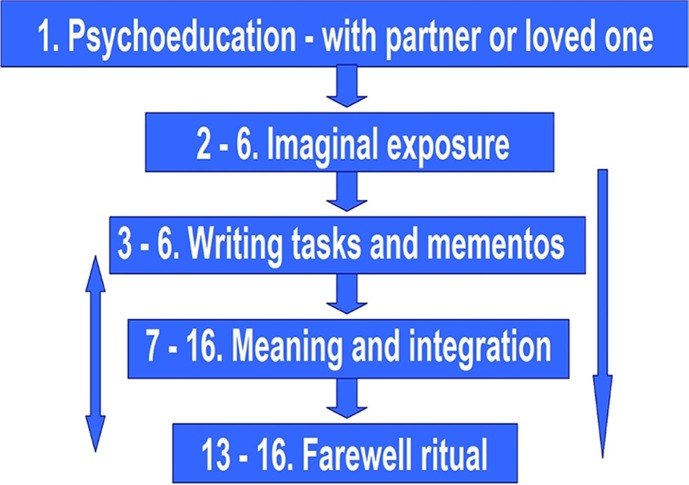


The overlapping numbers mean that the different elements of the therapy may take place simultaneously in the same session. Psychoeducation is the theme of the first session, but it is covered again in every element of the therapy. From session 2 onwards, each session starts with a brief review of the previous session and a conversation on how the PTSD symptoms are developing. The therapist then explains the structure of the current session and its rationale. Relaxation exercises are carried out prior to exposure. During exposure, the patient's eyes are closed. If sadness is expressed during the “hot spot” (Nijdam, Baas, Olff, & Gersons, 2013), the therapist has the patient open his or her eyes again and the session is discussed. The exposure lasts 15–20 minutes. Five sessions are generally sufficient for the exposure part of the treatment. More sessions may be needed if the patient does not feel safe enough with the therapist, or if feelings of shame and/or guilt keep the patient from expressing emotions. It is also possible for patients to have a great deal of difficulty with emotions in general; they may complain that they hardly feel anything at all. The exposure stage is finished when there are no longer any overly intense emotions associated with the traumatic experiences. This means that when the events are discussed later in the therapy, the patient is no longer overwhelmed by the associated emotions.

From the third session onward, the mementos (“memory aids”) are involved in the exposure. The significance of the ongoing letter is explained. From that point on, the patient will try to find a quiet moment to write for half an hour every day. He or she will read (parts of) the letters aloud during subsequent sessions. The emphasis each time is on expressing emotions that are evoked by the letters. The primary focus of the writing is rage and anger, and sometimes also hate or death wishes directed at others. The writing task is done when all rage has been expressed. Other types of letters may also be included, such as farewell letters in the context of grief. Sadness and feelings of abandonment play a major role in those letters. At some time around session 7, the meaning and integration stage is introduced. The duration of this stage may vary, depending on the person involved and their experiences.

Around the 13th session, it is time to start talking about rounding-off the therapy. The therapist explains the farewell ritual again and encourages the patient to come up with a form of farewell that is appropriate to his or her personal framework and to the traumatic event. For example, the patient might burn the letters in the presence of a loved one. The patient can then celebrate the end of the therapy and the leaving behind of the trauma with a nice dinner. As a rule, the therapist does not participate in the farewell ritual. The ritual also implies a farewell to the therapist and a reunification with the loved ones.

Before ending the therapy, the therapist needs to ensure that the symptoms of PTSD have actually abated. Checklists that systematically assess PTSD symptoms can be helpful here. If the symptoms are still present, the therapist needs to find out what is still maintaining the symptoms. This may mean that parts of the protocol have to be repeated. It is customary to conduct a follow-up evaluation about 6 months after termination of treatment to see whether the therapy has achieved stable long-term results.

BEPP is an eclectic treatment protocol in which different elements from a set of diverse psychotherapy approaches have been brought together in specific order. Psychoeducation comes from cognitive therapy while the imaginary exposure has, as its background, elements from CBT and from hypnotherapy. The use of memorabilia as the application of a farewell ritual has its roots in directive psychotherapy. The letter writing has become well-known in grief treatment. The meaning and integration is based on a psychodynamic treatment.

For therapists trained in a specific school of psychotherapy, it can be difficult to adapt and understand the more differentiated BEPP protocol. In daily practice, therapists who are used to the application of CBT or EMDR, often pay attention to the expression of emotions and to meaning issues, thus deviating from the treatment protocols. BEPP is often praised as a complete and comprehensive protocol with a natural process.

BEPP has been developed especially for the treatment of PTSD. This means that comorbidities such as trauma related depression and substance related disorders are not contraindications. However, severe depression and psychosis are judged as contraindication for BEPP. It is necessary to be able to feel and express emotions. Benzodiazepines therefore are a handicap in BEPP treatment, while the use of antidepressants isn't. In clinical practice, BEPP has also been used in the treatment of complex PTSD.

## Scientific evidence on BEPP treatment

The protocol was developed in the 1980s and 1990s to treat PTSD in police officers. Randomized controlled research showed that BEPP was effective in police officers (Gersons, Carlier, Lamberts, & Van der Kolk, 2000). It was noted in this initial study that there was no difference between the group receiving therapy and the control group after four sessions, but that there was a difference after the therapy was completed. A follow-up assessment after 3 months showed that the difference between the treated and non-treated group had become even greater; the treated group continued to show further improvement.

The effectiveness of a form of treatment is expressed in what is known as an “effect size.” In a meta-analysis, Bradley, Greene, Russ, and Westen (2005) calculated an effect size of 1.30 for BEPP. The average effect size of all active treatments for PTSD was 1.43. Lindauer et al. (2005a) showed that BEPP was effective not only in police officers, but also in a more general population of PTSD patients with various sorts of traumas, achieving an effect size of 1.62. This is comparable to the effect size of the best forms of CBT in the meta-analysis mentioned above (Bradley et al., 2005). These findings were also confirmed in a recent study on the effectiveness of BEPP in comparison with EMDR (Nijdam, Gersons, Reitsma, de Jongh, & Olff, 2012). BEPP and EMDR showed equal effectiveness in this study but EMDR reached the result somewhat earlier. Lindauer also showed significant improvements in biological parameters (Lindauer et al. 2004, 2005b), particularly in heart rate. The frontal lobes also appeared to be less inhibited after BEPP. Only the limbic system remained more sensitive.

A replication study, conducted by an independent research group in Switzerland, confirmed that BEPP was effective in decreasing PTSD symptoms as well as comorbid depression and anxiety. In addition, BEPP appeared to stimulate posttraumatic growth (Schnyder, Müller, Maercker, & Wittmann, 2011). Another replication study is currently being carried out in Lithuania.

## What is learned in BEPP from traumatic experiences?

The first learning point in BEPP is to see and understand during psychoeducation the relationship between the traumatic experience and the subsequent development of the symptoms of PTSD. This way, the patient learns that the origins of everyday problems such as being too easily irritated or having difficulties with sleep are not necessarily attributable to their character or to others around the patient or to other problems (which is possible of course). The main learning point then is that PTSD is a mode that is needed and adaptive when facing danger but is dysfunctional when danger has disappeared. People start to understand that their behavior is still shaped in a way of defense or attack. This is the first cognitive step to take in the treatment of PTSD.

Also, when a partner accompanies the patient to the first session, he or she will learn that their next of kin also suffer from their dysfunctional state. Partners often try to adapt to the patient suffering from PTSD. Children are asked not to disturb or understand why father or mother will not go to a birthday party. Here again, family problems will be reframed, and understood as related to the traumatic experiences.

The key learning point in BEPP, however, is that the fear that “it will happen again” is in essence a fear that the emotions of sadness and fury related to the traumatic event may be too strong to handle. Patients start to realize that they constantly try to control and suppress the emotions connected to the bad memories. In comparison with other trauma-focused treatments patents, are not repeatedly exposed to the traumatic experience to decrease the anxiety. They are also not asked to rate the level of anxiety and to see if it decreases by repeated confrontation and/or distraction. In BEPP, patients are asked to feel and accept how sad they are about what happened to them and how much anger this has brought about. Once the emotions are accessed, felt and expressed in a cathartic way, patients will feel relief. In essence, patients learn to feel and accept the value of emotions.

In the next phase of the domain of meaning, this opening up for emotions like sadness and grief will also increase patients’ interest in how their view of the world has changed, and that they themselves have changed or need to adapt to a new view of themselves and the world. In BEPP, this is taken together in the English slogan “sadder but wiser”. This slogan wonderfully summarizes BEPP. First, one has to experience the strong emotions after which the learning about life will start. This can be posttraumatic growth but it can also be learning about one's childhood and how it shaped one's expectations about the world. Often, in trauma-focused treatments, therapists try to help their patients “feeling good,” “I am strong,” “I can stand it,” “I am OK,” “I am not guilty,” “I can respect myself,” and so on. Without any doubt, such cognitions frequently signify an important improvement during treatment. In BEPP, such cognitions will also be part of the learning process. But in BEPP we put much more emphasis on the vulnerability of human beings, so that patients learn that, for example, life is actually not so easy, that we should not trust just everyone, and that sometimes we need to accept that we feel guilty.

In BEPP, as in crisis theory, trauma is also seen as an opportunity, albeit unwelcome and aversive, to learn about the world and about oneself.

## Footnotes

1 The BEPP protocol (2011) can be obtained by emailing to b.p.gersons@amc.nl or contact BEPP on their website: www.traumatreatment.eu. It is available in English, German, Dutch, and Lithuanian.

## References

[CIT0001] Bradley R, Greene J, Russ E, Westen D (2005). A multidimensional meta-analysis of psychotherapy for PTSD. American Journal of Psychiatry.

[CIT0002] Foa E. B, Keane T. M, Friedman M. J (2000). Effective treatments for PTSD.

[CIT0003] Foa E. B, Rothbaum B. O (1998). Treating the trauma of rape: Cognitive-behavioral therapy for PTSD.

[CIT0004] Fromm E, Shor R. E (1979). Hypnosis: Developments in research and new perspectives.

[CIT0005] Gersons B. P. R (1989). Patterns of posttraumatic stress disorder among police officers following shooting incidents: The two-dimensional model and some treatment implications. Journal of Traumatic Stress.

[CIT0006] Gersons B. P. R, Carlier I. V. E, Lamberts R. D, Van der Kolk B (2000). A randomized clinical trial of brief eclectic psychotherapy in police officers with posttraumatic stress disorder. Journal of Traumatic Stress.

[CIT0007] Gersons B. P. R, Olff M (2005). Treatment strategies for PTSD.

[CIT0008] Lindauer R. J. L, Gersons B. P. R, Van Meijel E. P. M, Blom K, Carlier I. V. E, Vrijlandt I (2005a). Effects of brief eclectic psychotherapy in patients with posttraumatic stress disorder: Randomized clinical trial. Journal of Traumatic Stress.

[CIT0009] Lindauer R. J. L, Vlieger E. J, Jalink M, Olff M, Carlier I. V. E, Majoie C. B. M. L (2004). Smaller hippocampal volume in Dutch police officers with posttraumatic stress disorder. Biological Psychiatry.

[CIT0010] Lindauer R. J. L, Vlieger E. J, Jalink M, Olff M, Carlier I. V. E, Majoie C. B. M. L (2005b). Effects of psychotherapy on hippocampal volume in out-patients with post-traumatic stress disorder: A MRI investigation. Psychological Medicine.

[CIT0011] Lindy J. D (1988). Vietnam: A casebook.

[CIT0012] Nijdam M. J, Baas M. A, Olff M, Gersons B. P. R (2013). Hotspots in trauma memories and their relationship to successful trauma-focused psychotherapy: A pilot study. Journal of Traumatic Stress.

[CIT0013] Nijdam M. J, Gersons B. P. R, Reitsma J. B, De Jongh A, Olff M (2012). Brief eclectic psychotherapy versus eye movement desensitization and reprocessing therapy in the treatment of posttraumatic stress disorder: Randomized controlled. British Journal of Psychiatry.

[CIT0014] Ramsay R. W (1977). Behavioural approaches to bereavement. Behaviour Research and Therapy.

[CIT0015] Schnyder U, Müller J, Maercker A, Wittmann L (2011). Brief eclectic psychotherapy for PTSD: A randomized controlled trial. Journal of Clinical Psychiatry.

[CIT0016] Shapiro F (1995). Eye movement desensitization and reprocessing.

[CIT0017] Van der Hart O (1985). Coping with loss: The therapeutic use of leave-taking rituals.

